# Polychlorinated Biphenyl and Organochlorine Pesticide Concentrations in Maternal Mid-Pregnancy Serum Samples: Association with Autism Spectrum Disorder and Intellectual Disability

**DOI:** 10.1289/EHP277

**Published:** 2016-08-23

**Authors:** Kristen Lyall, Lisa. A. Croen, Andreas Sjödin, Cathleen K. Yoshida, Ousseny Zerbo, Martin Kharrazi, Gayle C. Windham

**Affiliations:** 1 Environmental Health Investigations Branch, California Department of Public Health, Richmond, California, USA; 2 Division of Research, Kaiser Permanente, Oakland, California, USA; 3 Division of Laboratory Sciences, National Center for Environmental Health, Centers for Disease Control and Prevention, Atlanta, Georgia, USA

## Abstract

**Background:**

Polychlorinated biphenyls (PCBs) and organochlorine pesticides (OCPs) are neurodevelopmental toxicants, but few studies have examined associations with autism spectrum disorder (ASD).

**Objectives:**

We aimed to determine whether prenatal exposure to PCBs and OCPs influences offspring risk of ASD and intellectual disability without autism (ID).

**Methods:**

We conducted a population-based case–control study among Southern California births, including children with ASD (*n* = 545) meeting *Diagnostic and Statistical Manual of Mental Disorders, 4th Edition* (DSM-IV-TR) criteria and ID (*n* = 181), as well as general population (GP) controls (*n* = 418). Concentrations of 11 PCB congeners and 2 OCPs measured in banked second-trimester serum samples were compared between the diagnostic groups. Logistic regression was used to calculate crude and adjusted odds ratios (AOR) for associations with ASD, and separately for ID, compared with GP controls, by quartiles of analyte concentrations in primary analyses.

**Results:**

Geometric mean levels of several PCB congeners were higher in the ASD group than in the ID and GP groups. ASD risk was elevated for a number of PCB congeners, particularly for the highest vs. lowest quartile of PCB138/158 (AOR = 1.79; 95% CI: 1.10, 2.71) and PCB153 (AOR = 1.82; 95% CI: 1.10, 3.02), and for highest deciles of other congeners in secondary analyses. PCB138/158 was also associated with increased ID (AOR = 2.41; 95% CI: 1.18, 4.91), though no trend was suggested. OCPs were not associated with increased risk of ASD in primary analyses, whereas nonmonotonic increases in risk of ID were found with *p,p*´-DDE.

**Conclusions:**

Our results suggest higher levels of some organochlorine compounds during pregnancy are associated with ASD and ID.

**Citation:**

Lyall K, Croen LA, Sjödin A, Yoshida CK, Zerbo O, Kharrazi M, Windham GC. 2017. Polychlorinated biphenyl and organochlorine pesticide concentrations in maternal mid-pregnancy serum samples: association with autism spectrum disorder and intellectual disability. Environ Health Perspect 125:474–480; http://dx.doi.org/10.1289/EHP277

## Introduction

Autism spectrum disorder (ASD) is a neurodevelopmental condition defined by presence of restricted, repetitive behaviors and deficits in social communication. Intellectual disability, a condition commonly co-occurring in ASD, is defined by impairments in general mental abilities and adaptive functioning (American Psychiatric Association 1994). Evidence supports a role for both genetic and environmental factors in the etiology of ASD and neurodevelopment more broadly (Antonelli et al. 2016; Hallmayer et al. 2011; Newschaffer et al. 2007), and emerging work points to environmental toxicants that have the ability, either directly or indirectly, to alter neurodevelopment during critical time periods in gestation (Braun et al. 2014; Cheslack-Postava et al. 2013; Grandjean and Landrigan 2014; Lyall et al. 2014; Rice and Barone 2000). One such class of toxicants is the organochlorine compounds (OCCs). These chemicals have the ability to cross the placenta (Wolff and Landrigan 2002) and influence a number of pathways previously implicated in ASD and neurodevelopment, including endocrine disruption, and effects on immune, reproductive, and nervous systems (Bell 2014; Chevrier et al. 2008; Hertz-Picciotto et al. 2008; Kimura-Kuroda et al. 2007; Shelton et al. 2012).

Polychlorinated biphenyl ethers (PCBs) and organochlorine pesticides (OCPs) are two classes of OCCs that have been designated as persistent organic pollutants (POPs). As a result of health concerns, these chemicals were banned in the 1970s in the United States and other countries. Due to their lipophilic nature, OCCs have bioaccumulated in the food chain, and levels are still measurable in blood samples today, including those from pregnant women, as well as in breast milk samples (Sjödin et al. 2014; Woodruff et al. 2011). Currently, the primary source of exposure to the general population in the United States is through the consumption of fatty foods (CDC 2009).

Although both PCBs and OCPs have known adverse effects on neurodevelopment (Korrick and Sagiv 2008; Ribas-Fitó et al. 2001; Rosas and Eskenazi 2008), only a few studies have examined prenatal exposure in association with risk of ASD specifically. A nonsignificant association for total prenatal PCB exposure was suggested in one small pilot study (Cheslack-Postava et al. 2013), while conflicting findings were reported for gestational exposure to PCBs and other endocrine-disrupting chemicals in association with autistic behaviors in another small study (Braun et al. 2014). A few additional studies have more indirectly suggested associations between ASD and exposure to these chemicals, as estimated by parental occupations, proximity to agricultural organochlorine pesticide use, or postnatal exposures (Felicetti 1981; Roberts et al. 2007; Rossignol et al. 2014; Windham et al. 2013). A number of investigations have examined OC exposure in association with a range of cognitive and developmental outcome measures, as reviewed elsewhere (Jurewicz et al. 2013), though work examining the diagnosis of ID (intellectual disability without autism) is limited. Thus, sufficiently powered studies examining ASD and ID risk in association with OCC exposure using biosamples collected during developmentally relevant time periods are lacking, leading us to conduct the current analyses.

## Methods

### Study Population

Details of the Early Markers for Autism (EMA) study have been previously described (Croen et al. 2008). Briefly, EMA is a population-based case–control study designed to identify biologic markers of autism that uses archived prenatal and newborn blood samples from California statewide screening programs. The EMA study population was drawn from live-born children in three counties from Southern California (Orange, San Diego, and Imperial) in 2000–2003 whose mothers were enrolled in the state’s Expanded Alphafetoprotein Prenatal Screening Program. Only mother–child pairs for whom both maternal prenatal and newborn screening blood specimens were available were eligible for inclusion. Study activities were approved by the California State Committee for the Protection of Human Subjects; it was determined at the CDC that the agency was not engaged in human subjects research.

Information from the California Department of Developmental Services (DDS), which provides services to individuals with autism, intellectual disability, and other developmental disabilities, was used to create three study groups: children with ASD, children with ID, and general population (GP) controls. ID cases did not include those of known etiology (e.g., chromosomal abnormalities). Following exclusion of DDS clients, GP controls were randomly sampled from prenatal screening program linked birth certificates, matching to ASD cases on sex, birth month, and birth year.

### Diagnostic Verification

The records of children with ASD and ID identified from DDS were obtained and reviewed by trained medical record abstractors. Following a protocol originally developed by the Metropolitan Atlanta Developmental Disabilities Surveillance Program (Yeargin-Allsopp et al. 2003), final case status was determined by expert clinical review of these diagnostic and clinical data. A final study classification of ASD was given if DSM-IV-TR (*Diagnostic and Statistical Manual of Mental Disorders, 4th Edition, Text Revision*) criteria were met; ASD cases were further qualified by presence or absence of co-occurring intellectual disability (here defined as for final classification of ID, but in the presence of ASD symptoms, yielding 287 ASD children with comorbid intellectual disability, 221 ASD children without intellectual disability, and 37 ASD children who did not have information available to make this classification). Final classification of ID was based on standardized test score results found in records (composite score on cognitive and functional tests < 70) in the absence of ASD symptoms. Expert clinician review identified 132 individuals from among those originally identified as ID in the DDS system as meeting ASD criteria, and they were therefore reclassified as ASD cases. By definition no GP control children were being served by DDS.

### Specimens and Measurements

Maternal second-trimester specimens were retrieved from the California Department of Public Health’s Project Baby’s Breath (M. Kharrazi, P.I.) archives, which includes serum and blood cell pellet specimens collected for routine prenatal screening during 15–19 weeks gestation. Maternal specimens were collected in serum separator tubes by obstetrical care service providers and underwent testing within 7 days of collection at a central laboratory (median time, 3 days). After 1–2 days of refrigeration, leftover specimens were stored at –20°C. Consent forms for the screening program distributed at the time of the blood collection stipulated that specimens and results from prenatal testing could be used for research purposes given IRB approval.

PCB congeners (*n* = 37) and 9 persistent pesticides [hexachlorobenzene, β-hexachlorocyclohexane, γ-hexachlorocyclohexane, oxychlordane, *trans*-nonachlor, mirex, 2,2-bis(4-chlorophenyl)-1,1-dichloroethene (*p,p*´-DDE), 2,2-bis(4-chlorophenyl)-1,1,1-trichloroethane (*p,p*´-DDT), and 2-(4-chlorophenyl)-2-(2-chlorophenyl)- 1,1,1-trichloroethane (*o,p*´-DDT)] were measured in maternal serum samples. Determination of target analytes was performed by gas chromatography–​isotope dilution high resolution mass spectrometry (GC-IDHRMS) employing a Double Focusing Sector (ThermoFinnigan DFS, Bremen, Germany) instrument (Jones et al. 2012). Concentrations of analytes were reported as ng/g lipid weight (weight of serum lipids), corrected for background (e.g., subtraction of median contamination of blank samples in the same run as the unknowns; 3 blanks were included in every set of 30 samples). The total lipid concentration was calculated from total cholesterol and triglyceride concentration according to Phillips et al. (1989). Total cholesterol and triglyceride serum concentration was measured using an enzymatic reaction on a Roche ModP chemistry analyzer (Roche, Basel, Switzerland). Limits of detection (LOD values) were calculated for each individual sample.

### Statistical Analyses

These analyses included singleton births with final case status (see “Diagnostic Verification”) and PCB/OCP measurements available. Individuals missing all exposure biomarkers (due to insufficient sample; *n* = 5), and individuals censored due to laboratory error (failure of automation system; *n* = 33) were excluded, leaving a total of 1,144 individuals. This number includes 545 children with ASD, 181 children with ID without ASD, and 418 GP control children.

For each of the 37 PCBs and 9 OCPs measured, the percent of the study group with levels above the LOD was calculated. Only those analytes with at least 60% of the values above the LOD were included in further analysis (11 PCBs, 2 OCPs), as has been recommended and utilized in prior work of these and other POPs to avoid biased estimates in data using imputed values for measurements < LOD (Lubin et al. 2004; Sjödin et al. 2008; Windham et al. 2015). Participants with measurements < LOD had their values replaced with LOD divided by the square root of 2 (Axelrad et al. 2009; Sjödin et al. 2014), although alternative methods for imputing values below the LOD were also utilized (see “Sensitivity Analyses,” below). Descriptive statistics, including calculation of geometric means, for PCBs and the two OCPs were compared among the ASD, ID, and GP groups, and correlation coefficients between congeners and pesticides were calculated. Potential covariates, obtained from birth certificates or screening program records, were selected on the basis of *a priori* associations with ASD/ID and/or the PCB/OCPs and were then examined for associations with ASD and ID and with the quartiles of analytes. Variables included in final adjusted models were the study matching factors (child sex, month and year of birth), maternal age, maternal race/ethnicity, maternal weight at time of sample collection, and parity. Other variables tested but not included in final models (as they did not alter estimates) were paternal age, maternal birthplace, and child low birth weight and gestational age. In addition, we examined the effect of potential co-pollutant confounding by examining adjustment of individual PCB congeners for other congeners, the sum of PCBs, and for the pesticides separately. Further, we examined whether adjustment for a data-driven measure of genetic inheritance (Price et al. 2006), obtained using principal components (PCs) derived from principal components analysis of available genome-wide data, altered results. Specifically, the top two PCs (which accounted for the majority of variation) were included in secondary analyses (adjusted as for final models with the exception of race/ethnicity, accounted for by the PCs). Adjustment for PCs was not conducted for the ID–GP comparisons, because genetic information was not available for the ID group.

We created a variable for total PCB exposure by summing the individual concentrations of congeners with detection rates > 60% (see Table 1 for congener-specific detection rates); other sums (Axelrad et al. 2009; Wolff et al. 1997) were also examined in secondary analyses (see “Appendix” in the Supplemental Material for a list).

**Table 1:** Descriptive statistics*^a^* of analytes (ng/g lipid weight) in the study population (*n *= 1,144).

Compound	LOD range^*b*^	% above LOD	Interquartile range (ng/g)	Mean ± SD	Median	Geometric mean (95% CI)	NHANES geometric mean (95% CI)^*c*^
PCB28	0.5–49.7	84	6.1–41.7	30.65 ± 39.3	15.0	15.10 (14.0, 16.2)	4.99 (4.66, 5.35)
PCB99	0.4–3.0	63	0.92–2.1	1.76 ± 1.40	1.40	1.46 (1.41, 1.51)	4.35 (3.94, 4.81)
PCB118	0.4–3.0	86	1.5–3.6	2.98 ± 2.56	2.30	2.37 (2.28, 2.46)	No information
PCB138/158	0.4–3.6	96	3.6–9.9	7.79 ± 6.92	5.85	5.81 (5.55, 6.08)	15.3 (14.0, 16.8)
PCB153	0.4–7.3	98	4.5–13.3	10.28 ± 9.19	7.70	7.64 (7.30, 7.99)	19.7 (18.4, 21.1)
PCB170	0.4–3.6	88	1.5–4.7	3.69 ± 3.30	2.80	2.74 (2.61, 2.87)	5.14 (4.82, 5.48)
PCB180	0.4–6.5	98	3.5–11.4	9.01 ± 8.26	6.70	6.46 (6.15, 6.77)	14.2 (13.4, 15.0)
PCB187	0.4–2.1	78	1.0–3.6	3.05 ± 3.52	1.90	1.99 (1.88, 2.10)	4.12 (3.84, 4.41)
PCB194	0.4–2.5	70	0.9–2.7	2.22 ± 2.08	1.50	1.63 (1.55, 1.70)	2.45 (2.28, 2.64)
PCB196/203	0.4–2.2	77	0.92–3.1	2.45 ± 2.29	1.70	1.77 (1.68, 1.85)	2.46 (2.27, 2.66)
PCB199	0.4–2.1	69	0.78–2.7	2.32 ± 2.76	1.40	1.52 (1.44, 1.60)	2.63 (2.49, 2.79)
Sum of above PCBs	NA	NA	34.9–94.7	73.2 ± 54.4	57.9	57.70 (55.3, 60.2)	—
*p,p*´-DDE	1.8–83.0	99.9	131.0–477.5	601.7 ± 1,256	218.0	270.0 (253, 288)	305.0 (273, 341)^*d*^
*trans*-Nonachlor	1.5–8.1	61	3.1–7.3	6.04 ± 5.5	4.70	4.89 (4.72, 5.07)	17.0 (15.4, 18.7)^*d*^
Abbreviations: PCB, polychlorinated biphenyl; *p,p*´-,DDE, *p,p*´-dichlorodiphenyldichloroethene; LOD, limit of detection. ^***a***^Range, means, and median calculated after replacing values < LOD with the LOD divided by the square root of 2. ^***b***^Variation in LODs for given analytes was observed as LODs were determined for each individual based on sample volume and concentration in the sample. ^***c***^Estimates for all females from the CDC (2009). Values from years of sampling 2003–2004 are shown (for comparison to EMA study dates). NHANES geometric means for the ≥ 20 years age group were similar to those shown for females. ^***d***^Not calculated for females, but male value = 5.28 (< LOD–6.14).							

Quartiles of chemical concentrations were created based on the GP control distribution. Tests of trend for these analyses were conducted using a Wald test in separate adjusted models using the numerical quartile variable for analyte levels. In secondary analyses, we also explored alternate parameterizations of the distribution, including high and low deciles. Matching was broken in analyses in order to include all individuals (e.g., children originally identified with ID but later determined to be ASD) with measured OCCs. Unconditional logistic regression was used to obtain crude and multivariable adjusted odds ratios (OR) and 95% confidence intervals (CIs) for the association between the categorized exposure biomarkers and ASD or ID, in comparison to GP controls.

For analyses of risk of ASD, we also conducted stratified analyses to examine potential effect modification by child sex and main categories of maternal race/ethnicity (non-Hispanic white, Hispanic). Further, we conducted analyses examining whether ASD risk differed according to presence or absence of intellectual disability by modeling risk of ASD with and without ID relative to GP controls.

### Sensitivity Analyses

Sensitivity analyses conducted to assess robustness of results included *a*) excluding from the ASD group individuals originally served as ID in the DDS system, but reclassified as ASD following expert review (*n* = 132 of the 545 ASD cases); *b*) using conditional logistic regression analyses within the subset of matched ASD–GP pairs (*n* = 788) to examine the impact of breaking matching; *c*) removing those individuals with values < LOD; and *d*) considering alternate strategies for replacing values < LOD, including using laboratory-read values for low sample volumes originally censored to < LOD, and multiple imputation using SAS PROC MI (SAS Institute Inc. 2015). Data used to impute missing values included all OCCs assessed here, case status, and all covariates used in adjusted models.

## Results

A total of 11 PCB congeners [PCBs 28, 99, 118, 138/158, 153, 170, 180, 187, 194, 196/203, 199; congeners reported together (138/158 and 196/203) indicate co-elution on the chromatographic system, yielding a combined concentration] and 2 OC pesticides (*trans*-nonachlor and *p,p*´-DDE) were measured at levels above the LOD in > 60% of the study population. Geometric mean concentrations were generally somewhat lower for these chemicals than the concentrations among all females from the National Health and Nutrition Examination Survey (NHANES) (as well as compared with individuals ≥ 20 years of age from this survey) (CDC 2009), except for PCB28, which was somewhat higher (Table 1). Most PCB congeners were significantly and highly correlated; other correlations between the exposure biomarkers were weak to moderate (see Table S1).

A number of demographic factors were associated with both outcomes and exposure levels (Table 2; see also Table S2). Most notably, children with ASD were approximately four times as likely to be male than female (as were GP controls due to matching), have older parents, and have mothers with higher education, whereas the ID group had a higher proportion of Hispanics and individuals utilizing government insurance programs at delivery, compared with the other groups (Table 2). Generally, among GP controls, factors related to higher socioeconomic status (SES) were related to higher PCB levels, whereas those linked with lower SES were related to higher pesticide levels (see Table S2).

**Table 2:** Basic characteristics of the study population by case status.

Characteristic	ASD (*n*= 545)	ID (*n *= 181)	GP (*n *= 418)
Maternal age (mean ± SD)	29.9 ± 5.6*	27.2 ± 6.3	28.7 ± 5.4
Paternal age (mean ± SD)	35.7 ± 14.6	36.9 ± 21.2	34.0 ± 14.2
Child year of birth (mean ± SD)	2001 ± 1.0	2001 ± 1.0	2001 ± 1.0
No. of prenatal visits (mean ± SD)	15.1 ± 13.9	15.4 ± 16.0	13.8 ± 10.1
No. of total live births (mean ± SD)	1.8 ± 0.9*	2.3 ± 1.5	2.1 ± 1.0
Child birth weight in grams (mean ± SD)	3390.9 ± 608.7	3,009 ± 868	3385.0 ± 588.8
Child gestational age in days (mean ± SD)	274.3 ± 27.1*	268.2 ± 30.2	279.0 ± 36.1
Multiparous [*n* (%)]	298 (55)**	119 (66)	259 (62)
Maternal birth place [*n* (%)]	**
United States	272 (50)	79 (44)	202 (48)
Mexico	131 (24)	84 (46)	128 (31)
Other	142 (26)	18 (10)	88 (21)
Maternal race/ethnicity [*n* (%)]	**
Non-Hispanic white	192 (35)	32 (18)	138 (33)
Asian	82 (15)	9 (5)	45 (11)
Black, Pacific Islander, other	48 (9)	13 (7)	35 (8)
Hispanic	218 (40)	126 (70)	197 (47)
Missing	5 (1)	1 (0.6)	3 (0.7)
Maternal education [*n* (%)]	**
Less than high school	97 (18)	76 (42)	102 (24)
High school	118 (22)	50 (28)	113 (27)
Some college/college degree	222 (41)	42 (23)	143 (34)
Postgraduate	100 (18)	11 (6)	56 (13)
Missing	8 (1.5)	2 (1)	4 (1)
Maternal age ≥ 35 years [*n* (%)]	103 (19)**	22 (12)	52 (13)
Paternal age ≥ 35 years [*n* (%)]	229 (42)**	63 (35)	142 (34)
Prenatal care initiated ≥ 3 months gestation [*n* (%)]	101 (19)	47 (27)	81 (19)
Insurance status at delivery [*n* (%)]	**
Self and other	19 (3)	7 (4)	18 (4)
Private insurance	289 (53)	49 (27)	207 (49)
Government program	237 (44)	125 (69)	193 (46)
Child sex	**
Male	446 (82)	104 (57)	345 (83)
Female	99 (18)	77 (43)	73 (17)
Child preterm (< 37 weeks) [*n* (%)]	66 (13)**	37 (22)	44 (11)
Child low birth weight (< 2,500 g) [*n* (%)]	42 (8)**	40 (22)	21 (5)
Abbreviations: ASD, autism spectrum disorder; GP, general population controls; ID, intellectual disability (without ASD). *t-Test comparing ASD to GP controls significant (*p *< 0.05). **Chi-squared test comparing the categorical variable across all three diagnostic groups significant (*p* < 0.05). Missing categories were not included in chi-squared tests.			

Comparing the exposure biomarker levels across diagnostic groups, geometric mean levels of PCBs and *trans*-nonachlor were higher in ASD cases relative to GP controls. Levels of PCB28 and *p,p*´-DDE were higher, while levels of other exposure biomarkers were lower, in the ID group relative to the ASD and GP groups (see Table S3).

### Associations with ASD

In crude and fully adjusted models, higher levels of a number of PCBs were associated with increased risk of ASD (Table 3), particularly for PCBs 138/158, 153, 170, and 180 [adjusted odds ratios (AORs) ~ 1.5 or greater for the top quartile]. Adjusted associations were strongest for PCB138/158 and PCB153 (for highest quartile relative to the lowest quartile, AOR = 1.79; 95% CI: 1.10, 2.92 and AOR = 1.82; 95% CI: 1.10, 3.02, respectively). These congeners were highly correlated (*r* = 0.98), though PCB153 was also highly correlated with PCB170 and 180 (*r* = 0.93 and 0.94). When additionally adjusting for genetic ancestry using the PCs from genome-wide data (instead of race/ethnicity), estimates were generally somewhat attenuated, though increases in risk were still suggested for PCBs 138/158 and 153 (AOR = 1.54; 95% CI: 0.87, 2.71 and AOR = 1.72; 95% CI: 0.97, 3.07, respectively). The sum of PCB congeners demonstrated a moderately elevated risk of ASD for the top quartile (OR = 1.39; Table 3), though the association was not significant (either including genetic ancestry or not); results for other sums we examined were similar (results not shown). Tests of trend were significant only for PCB 138/158 and PCB153 (*p* = 0.03 and *p* = 0.04 and respectively). Elevations in risk of ASD with the two OC pesticides were not suggested in crude or adjusted models (Table 3).

**Table 3:** Prenatal PCB and OC pesticide levels (quartiles) in association with ASD risk (relative to GP controls).

Pesticide/quartile	Value (ng/g lipid)	*n *ASD cases	Unadjusted OR (95% CI)	AOR (95% CI)^*a*^
PCBs
PCB28
Q1	< 5.3	110	1.0 (referent)	1.0
Q2	5.3–< 14.15	143	1.22 (0.84, 1.76)	1.17 (0.79, 1.73)
Q3	14.15–< 37.1	144	1.27 (0.88, 1.84)	1.20 (0.81, 1.77)
Q4	≥ 37.1	148	1.29 (0.90, 1.87)	1.27 (0.87, 1.86)
PCB99
Q1	< 0.92	97	1.0	1.0
Q2	0.92–< 1.3	104	1.19 (0.79, 1.78)	1.12 (0.74, 1.70)
Q3	1.3–< 2.0	134	1.27 (0.87, 1.87)	1.08 (0.72, 1.62)
Q4	≥ 2.0	173	1.64 (1.13, 2.39)	1.17 (0.75, 1.83)
PCB118
Q1	< 1.4	79	1.0	1.0
Q2	1.4–< 2.3	133	1.44 (0.97, 2.14)	1.29 (0.86, 1.95)
Q3	2.3–< 3.6	142	1.70 (1.14, 2.54)	1.38 (0.90, 2.11)
Q4	≥ 3.6	152	1.71 (1.15, 2.54)	1.15 (0.72, 1.82)
PCB138/158
Q1	< 3.2	83	1.0	1.0
Q2	3.2–< 5.5	119	1.43 (0.97, 2.12)	1.39 (0.92, 2.10)
Q3	5.5–< 8.9	135	1.60 (1.08, 2.35)	1.34 (0.87, 2.07)
Q4	≥ 8.9	197	2.33 (1.60, 3.39)	1.79 (1.10, 2.92)
PCB153
Q1	< 4.2	87	1.0	1.0
Q2	4.2–< 7.4	117	1.35 (0.91, 1.99)	1.32 (0.88, 1.99)
Q3	7.4–< 11.7	128	1.45 (0.98, 2.13)	1.24 (0.80, 1.93)
Q4	≥ 11.7	204	2.33 (1.60, 3.37)	1.82 (1.10, 3.02)
PCB170
Q1	< 1.5	88	1.0	1.0
Q2	1.5–< 2.6	112	1.27 (0.85, 1.90)	1.15 (0.76, 1.76)
Q3	2.6–< 4.3	133	1.39 (0.94, 2.05)	1.17 (0.75, 1.83)
Q4	≥ 4.3	183	1.97 (1.35, 2.89)	1.48 (0.88, 2.50)
PCB180
Q1	< 3.4	95	1.0	1.0
Q2	3.4–< 6.1	110	1.09 (0.74, 1.61)	1.00 (0.66, 1.50)
Q3	6.1–< 10.4	139	1.41 (0.96, 2.06)	1.17 (0.75, 1.81)
Q4	≥ 10.4	195	1.97 (1.37, 2.85)	1.49 (0.89, 2.49)
PCB187
Q1	< 0.92	97	1.0	1.0
Q2	0.92–< 1.8	94	0.98 (0.65, 1.47)	0.89 (0.58, 1.36)
Q3	1.8–< 3.3	144	1.42 (0.97, 2.09)	1.22 (0.79, 1.87)
Q4	≥ 3.3	174	1.74 (1.19, 2.53)	1.32 (0.79, 2.20)
PCB194
Q1	< 0.9	101	1.0	1.0
Q2	0.9–< 1.5	105	1.05 (0.71, 1.56)	0.99 (0.65, 1.49)
Q3	1.5–< 2.6	129	1.26 (0.86, 1.86)	1.06 (0.68, 1.63)
Q4	≥ 2.6	170	1.65 (1.13, 2.40)	1.24 (0.76, 2.03)
PCB196/203
Q1	< 0.92	107	1.0	1.0
Q2	0.92–< 1.6	83	0.86 (0.57, 1.29)	0.79 (0.52, 1.21)
Q3	1.6–< 2.9	147	1.26 (0.86, 1.82)	1.05 (0.69, 1.61)
Q4	≥ 2.9	172	1.56 (1.08, 2.26)	1.13 (0.68, 1.88)
PCB199
Q1	< 0.72	101	1.0	1.0
Q2	0.72–< 1.3	96	1.04 (0.69, 1.55)	0.93 (0.61, 1.41)
Q3	1.3–< 2.6	153	1.46 (1.00, 2.12)	1.17 (0.76, 1.80)
Q4	≥ 2.6	159	1.59 (1.09, 2.32)	1.13 (0.68, 1.89)
Sum of above PCBs
Q1	< 33.4	94	1.0	1.0
Q2	33.4–< 55.3	119	1.27 (0.86, 1.87)	1.08 (0.72, 1.63)
Q3	55.3–< 86.3	115	1.22 (0.83, 1.81)	0.99 (0.64, 1.51)
Q4	≥ 86.3	172	1.83 (1.25, 2.67)	1.36 (0.88, 2.11)
OC pesticides
*p,p*´-DDE
Q1	< 121.7	114	1.0	1.0
Q2	121.7–< 212.5	164	1.43 (0.99, 2.05)	1.35 (0.93, 1.96)
Q3	212.5–< 505.4	145	1.27 (0.88, 1.83)	1.16 (0.77, 1.74)
Q4	≥ 505.4	122	1.06 (0.73, 1.54)	0.90 (0.57, 1.42)
*trans*-Nonachlor
Q1	< 3.1	114	1.0	1.0
Q2	3.1–< 4.7	120	1.01 (0.69, 1.48)	0.92 (0.62, 1.36)
Q3	4.7–< 7.1	127	1.06 (0.72, 1.55)	0.84 (0.56, 1.26)
Q4	≥ 7.1	146	1.22 (0.83, 1.77)	0.84 (0.55, 1.28)
Abbreviations: AOR, adjusted odds ratio; ASD, autism spectrum disorder; CI, confidence interval; *p,p*’-DDE, *p,p*’-dichlorodiphenyldichloroethene; GP, general population controls; OR, odds ratio; PCB, polychlorinated biphenyl; OC, organochlorine; Q1–Q4, quartiles 1–4. ^***a***^Adjusted for matching factors (child sex, month and year of birth), maternal age (continuous), maternal race/ethnicity (non-Hispanic white, Asian, black/Pacific Islander/or other, Hispanic, or missing), maternal weight at time of sample collection (quartiles), parity (multi- vs. primiparous), and maternal education (< high school, high school, college, graduate). Addition of other covariates listed in text, or adjustment for only maternal age and parity, yielded similar results.				

Due to the high correlation between most PCB congeners, results of many co-pollutant models yielded estimates with wide confidence intervals; nonetheless, adjustment for moderate or weakly correlated chemicals did not alter findings (data not shown). Examination of greater extremes of the distribution suggested higher levels (top decile relative to the lowest decile) increased risk further for some congeners, including some not suggested in primary quartile analyses (particularly PCBs 28, 180, 187, and 196/203), though this pattern was not uniform across congeners (see Table S4). These analyses were also more suggestive of increases in risk of ASD, with levels of *trans*-nonachlor above the lowest decile (with highest risk observed for the 10th–25th percentile, rather than for the top decile).

Despite some variation in results stratified by gender and race/ethnicity and according to ASD with and without ID, no clear patterns across strata emerged, and overall, similar associations as those seen in primary analyses were suggested (see Table S5). Sensitivity analyses also generally supported primary results (see Table S6). Removing individuals with values < LOD had no impact on the results (data not shown), nor did excluding ASD cases originally identified as ID.

### Associations with ID

In analyses of risk of ID relative to GP controls, inverse associations were seen for a number of PCB congeners (PCBs 118, 153, 170, 187, 194, 196/203, and 199) in crude models (Table 4). However, in fully adjusted models that accounted for confounding by demographic factors, many associations reversed in direction, and AORs elevated above 1.5 were seen for the highest levels of PCBs 138/158, 153, 187, and 196/203. Confidence intervals were wide in these analyses due to the smaller number of ID cases. The strongest adjusted associations noted were for increases in ID risk with the 2nd and 4th quartiles of PCB138/158, though risk for those in the 3rd quartile was not increased (*p* for trend = 0.17). The sum of PCBs demonstrated a moderate, though nonsignificant, elevation in risk of ID for the top quartile, similar to that seen for ASD. Of the OC pesticides, the 3rd quartile of *p,p*´-DDE showed a significantly increased risk of ID in crude and adjusted models (AOR relative to 1st quartile = 1.89; 95% CI: 1.03, 3.44), though no trend was suggested (*p* for trend = 0.57) and the 4th quartile did not indicate elevated risk. Adjusted analyses examining the highest decile of levels of these chemicals (see Table S4) were more suggestive of increased risk of ID with higher exposure, particularly for PCBs and the same congeners suggested in association with ASD, with many of these associations statistically significant, though confidence intervals were quite wide.

**Table 4:** Prenatal PCB and OC Pesticide levels in association with ID risk (relative to GP controls).

Pesticide/quartile	ID *n*	Unadjusted OR (95% CI)	AOR (95% CI)^*a*^
PCBs
PCB28
Q1	46	1.0 (referent)	1.0
Q2	31	0.63 (0.37, 1.07)	0.68 (0.38, 1.22)
Q3	47	0.99 (0.61, 1.62)	1.01 (0.59, 1.75)
Q4	57	1.19 (0.74, 1.92)	1.00 (0.59, 1.68)
PCB99
Q1	39	1.0	1.0
Q2	52	1.48 (0.89, 2.45)	1.48 (0.85, 2.59)
Q3	41	0.97 (0.58, 1.63)	1.17 (0.66, 2.08)
Q4	28	0.66 (0.38, 1.16)	1.35 (0.68, 2.68)
PCB118
Q1	45	1.0	1.0
Q2	59	1.12 (0.69, 1.81)	1.44 (0.84, 2.48)
Q3	32	0.67 (0.39, 1.15)	0.88 (0.47, 1.65)
Q4	24	0.47 (0.27, 0.84)	0.87 (0.42, 1.81)
PCB138/158
Q1	50	1.0	1.0
Q2	62	1.24 (0.78, 1.97)	2.29 (1.32, 3.97)
Q3	27	0.53 (0.31, 0.91)	0.98 (0.52, 1.85)
Q4	37	0.73 (0.44, 1.20)	2.41 (1.18, 4.91)
PCB153
Q1	62	1.0	1.0
Q2	58	0.93 (0.59, 1.45)	1.55 (0.92, 2.62)
Q3	26	0.41 (0.24, 0.69)	0.77 (0.41, 1.44)
Q4	35	0.55 (0.34, 0.91)	1.76 (0.87, 3.57)
PCB170
Q1	60	1.0	1.0
Q2	39	0.65 (0.40, 1.07)	0.93 (0.53, 1.64)
Q3	31	0.48 (0.28, 0.80)	0.88 (0.47, 1.64)
Q4	30	0.48 (0.28, 0.80)	1.42 (0.69, 2.95)
PCB180
Q1	69	1.0	1.0
Q2	44	0.60 (0.38, 0.96)	0.92 (0.54, 1.57)
Q3	39	0.54 (0.34, 0.88)	0.97 (0.53, 1.74)
Q4	29	0.40 (0.24, 0.68)	1.35 (0.64, 2.81)
PCB187
Q1	55	1.0	1.0
Q2	39	0.72 (0.43, 1.18)	1.11 (0.63, 1.95)
Q3	38	0.66 (0.40, 1.09)	1.16 (0.63, 2.11)
Q4	28	0.49 (0.29, 0.84)	1.68 (0.79, 3.58)
PCB194
Q1	58	1.0	1.0
Q2	46	0.80 (0.50, 1.30)	1.05 (0.62, 1.80)
Q3	31	0.53 (0.31, 0.89)	0.81 (0.44, 1.49)
Q4	25	0.42 (0.24, 0.73)	1.20 (0.57, 2.50)
PCB196/203
Q1	52	1.0	1.0
Q2	49	1.04 (0.64, 1.69)	1.51 (0.87, 2.62)
Q3	32	0.56 (0.33, 0.95)	1.00 (0.53, 1.88)
Q4	27	0.50 (0.29, 0.87)	1.54 (0.71, 3.31)
PCB199
Q1	54	1.0	1.0
Q2	47	0.95 (0.58, 1.54)	1.27 (0.74, 2.18)
Q3	34	0.61 (0.36, 1.01)	0.98 (0.53, 1.80)
Q4	25	0.47 (0.27, 0.81)	1.36 (0.64, 2.92)
Sum of above PCBs
Q1	46	1.0	1.0
Q2	35	0.76 (0.45, 1.28)	1.00 (0.56, 1.78)
Q3	40	0.87 (0.52, 1.45)	1.21 (0.68, 2.15)
Q4	39	0.85 (0.51, 1.42)	1.43 (0.79, 2.58)
OC pesticides	
*p,p*´-DDE
Q1	31	1.0	1.0
Q2	45	1.44 (0.85, 2.45)	1.42 (0.80, 2.54)
Q3	62	2.00 (1.20, 3.33)	1.89 (1.03, 3.44)
Q4	43	1.37 (0.80, 2.34)	1.15 (0.59, 2.24)
*trans*-Nonachlor
Q1	53	1.0	1.0
Q2	36	0.65 (0.39, 1.09)	0.67 (0.38, 1.17)
Q3	33	0.59 (0.35, 0.99)	0.98 (0.54, 1.79)
Q4	38	0.68 (0.41, 1.13)	0.97 (0.53, 1.77)
Abbreviations: ID, Intellectual disability without autism; other abbreviations as for Table 3. ^***a***^Adjusted as in Table 3.			

## Discussion

The results of this large, population based case–control study suggest that exposure to PCB congeners *in utero* may influence risk of ASD in offspring. This is one of the few studies to date examining prenatal exposure to OCCs, with exposures assessed from biospecimens collected during pregnancy, in relation to ASD and ID diagnoses. Our findings add to potential neurodevelopmental concerns surrounding these chemicals.

Our results from a number of different modeling strategies overall suggested increased risk of ASD with higher levels of PCBs. Results were generally consistent for risk of ID, after accounting for strong confounding by demographic factors. Primary analyses highlighted PCBs 138/158 and 153 in association with ASD, though other correlated congeners also demonstrated associations above the null. PCB138/158 was also associated with over a doubling in risk of ID (though no dose–response trend was evident), perhaps suggesting the impact of exposure to this congener on neurodevelopment broadly. Associations with even higher levels (e.g., the top decile) of PCBs suggested potentially higher risks of ASD and ID for additional PCBs (particularly 28, 180, 187, and 196/203), though we had limited statistical power to fully address this question for analyses of ID. Both PCB138/158 and PCB153 are among the most frequently detected PCBs (Sjödin et al. 2014; Woodruff et al. 2011); structurally, they are both non-dioxin-like, and we did find evidence for elevated risks with some other non-dioxin-like congeners (which include PCBs 28, 99, 170, 180, 187, 194, 196/203, and 199) in analyses of higher extremes of the distribution. PCB153 is also a cytochrome p450 (CYP)-inducing PCB (Wolff et al. 1997)—of interest given the role of CYP p450 genes in the breakdown of chemicals and the observation that genes in this family have been implicated in ASD (Chakrabarti et al. 2009; Veatch et al. 2015). This and other PCB congeners have been previously associated with low birth weight (Casas et al. 2015) and disruption of thyroid hormones (Liu et al. 2012), relevant to both ASD and ID. In one of the two previous studies examining risk of ASD or ASD symptoms in relation to measured prenatal levels of PCBs, a suggestive association with overall PCBs measured during pregnancy and ASD was found (Cheslack-Postava et al. 2013). In the other study, an inverse association between PCB178 but also a positive association with PCB153 were reported with autistic behaviors as measured by the Social Responsiveness Scale (Braun et al. 2014). These prior studies may have been influenced by limited detection frequency (in the latter) and/or small sample size. Overall, however, results across our work and these studies are suggestive of an association between higher levels of PCBs and risk of ASD, and possibly also ID.

We did not find evidence that higher levels of prenatal exposure to *p,p*´-DDE and *trans*-nonachlor increased risk of ASD, though decile analyses did suggest increases in risk and potential nonlinear trends with *trans*-nonachlor that should be further explored. Other OCPs had too low a detection frequency to be included in our primary analyses, limiting our ability to report on associations with levels of these chemicals. Individuals in the third quartile of *p,p*´-DDE levels had significantly increased odds of ID, but no dose–response effect was suggested. In previous work, an increased risk of autism in association with estimated maternal exposure to two other OCPs (dicofol and endosulfan) was reported (Roberts et al. 2007). Multiple studies have reported significant reductions in cognitive and neurodevelopmental scores (Torres-Sánchez et al. 2013) and various other neurodevelopmental deficits with prenatal DDE exposure (Rosas and Eskenazi 2008), and though findings are not consistent, animal studies have provided a biological basis for disruption of endocrine and immune systems, and evidence of neurotoxicity with prenatal exposure to OCPs (Grandjean and Landrigan 2014). Thus, continued investigation of OCPs in association with ASD and ID is warranted.

Our work suggests that ASD and ID may share some OCC risk factors. We did not note large differences in risk when examining ASD with or without co-occurring intellectual disability, though further consideration of how risk may differ between ASD, ID, and ASD comorbid with ID, may prove fruitful in identifying risk factors specific to ASD symptomatology versus factors related to impaired neurodevelopment more broadly.

We did not see strong increases in risk of either ASD or ID with higher levels of the sum of all PCB congeners detected or with other, more mechanistically or functionally based PCB sums. This lack of association with combined congeners may suggest that individual congeners play a greater role in associations with neurodevelopmental outcomes, or that simple summation does not adequately represent combined effects of mixtures of chemicals. Additional methodologically focused studies are needed to examine the effects of exposure to multiple chemicals.

This study has a number of strengths that represent significant improvements over prior work. These include a large sample size, measured levels of OCCs during gestational time periods critical to neurodevelopment, ability to adjust for a number of confounders and explore potential modifiers, and ASD diagnoses confirmed by expert clinician review. However, certain limitations should be considered in weighing these results. Our ability to tease out effects of correlated congeners was limited. Because people are exposed to multiple chemicals, and individual PCB congeners are highly correlated, attribution of risk to a given congener should be interpreted with caution. Our study had somewhat lower average levels of some PCB congeners than those reported in NHANES; geographic and demographic differences, including differences in age distributions, may account for this. Positive associations with PCBs were attenuated after accounting for genetic ancestry; thus, self-reported race/ethnicity information may not fully capture confounding that could be related to these factors.

As mentioned, multiple pathways could underlie associations between the OCCs examined here and ASD and ID. These include endocrine and/or immune system disruption, direct impacts on neuronal development, and epigenetic effects. PCBs and OCPs have been shown to alter thyroid hormone levels, and differences in neuronal development by PCB exposure through this pathway have been demonstrated *in vitro* (Kimura-Kuroda et al. 2007; Liu et al. 2012). Given extensive cross-talk among the developing immune and nervous systems (Bilbo and Schwarz 2012), established immune aberrations in autism (Goines et al. 2011; Mead and Ashwood 2015), and influence of OCCs on immune system development (Hertz-Picciotto et al. 2008), effects on the immune system is another particularly likely mechanism linking these factors.

## Conclusion

The overall pattern of our results suggests increases in risk of ASD and ID with prenatal exposure to higher levels of a number of OCCs, particularly PCBs. Future work should further consider genetic background in the role of these exposures on neurodevelopmental outcomes. Continued investigation of OCCs in association with ASD and ID is needed, given our findings and the dearth of studies investigating this topic.

## Supplemental Material

(216 KB) PDF
