# Supplementary material for: Polychlorinated Biphenyl and Organochlorine Pesticide Concentrations in Maternal Mid-Pregnancy Serum Samples: Association with Autism Spectrum Disorder and Intellectual Disability
Source: Environ Health Perspect. 2016 Aug 23;125(3):474–80. doi: 10.1289/EHP277 (PMC5332182; doi:10.1289/EHP277)
Supplement: (216 KB) PDF [file EHP277.s001.acco.pdf]

Environ Health Perspect
DOI: 10.1289/EHP277

Note to readers with disabilities: *EHP* strives to ensure that all journal content is accessible to all readers. However, some figures and Supplemental Material published in *EHP* articles may not conform to 508 standards due to the complexity of the information being presented. If you need assistance accessing journal content, please contact ehp508@niehs.nih.gov. Our staff will work with you to assess and meet your accessibility needs within 3 working days.

Supplemental Material

Polychlorinated Biphenyl and Organochlorine Pesticide Concentrations in Maternal Mid-Pregnancy Serum Samples: Association with Autism Spectrum Disorder and Intellectual Disability

Kristen Lyall, Lisa.A. Croen, Andreas Sjödin, Cathleen K. Yoshida, Ousseny Zerbo, Martin Kharrazi, and Gayle C. Windham

Table of Contents

Appendix: PCB congeners included in summary variables examined

- Sum of PCB congeners detected (primary, shown in tables)
- Sum of highly detected (≥90% above LOD) PCBs
- Sum of highly detected (≥80% above LOD) PCBs
- Sum of highly chlorinated PCBs
- Sum of non-dioxin-like PCBs
- Sum of cytochrome-p450 inducing PCBs

Table S1: Correlation across analytes in the study population

Table S2: Demographic and basic characteristics by quartiles of selected analytes in controls

Table S3: Geometric means by diagnostic status

Table S4: Association between the top decile of organochlorine chemicals and risk of ASD and ID

Table S5: Stratified and Subgroup Analyses of Prenatal PCB and OC Pesticide levels in association with ASI
(vs GP controls)

Table S6: Sensitivity Analyses of Prenatal PCB and OC Pesticide levels in association with ASD
(vs GP controls)

Appendix: PCB congeners included in summary variables examined[1]

Sum of PCB congeners detected (primary, shown in tables):

PCBs 28, 99, 118, 138/158, 153, 170, 180, 187, 194, 196/203, 199

Sum of highly detected (≥90% above LOD) PCBs:

PCBs 138/158, 153, 180

Sum of highly detected (≥80% above LOD) PCBs:

PCBs 28, 118, 138/158, 153, 180

Sum of highly chlorinated PCBs:

PCBs 170, 180, 187, 194, 196/203, 199

Sum of non-dioxin-like PCBs:

PCBs 28, 99, 138/158, 153, 170, 180, 187, 194, 196/203, 199

Sum of cytochrome-p450 inducing PCBs:

PCBs 153, 180, 196/203

[1]Note additional PCB congeners may fall within these groupings but were not detected at high enough frequencies in our study population to utilize in summary scores. Groupings were selected based on the literature.[27,29]

Table S1: Correlation across analytes in the study population

	PCB28	PCB99	PCB118	PCB138/ 158	PCB153	PCB170	PCB180	PCB187	PCB194	PCB196/ 203	PCB199	p,p'-DDE	Trans-Nonachlor
PCB28	1	0.15<br><.0001	0.05<br>0.09	0.03<br>0.31	0.01<br>0.66	0.01<br>0.63	0.007<br>0.82	0.04<br>0.22	0.04<br>0.20	0.03<br>0.33	0.03<br>0.29	-0.01<br>0.69	0.008<br>0.79
PCB99		1	0.87<br><.0001	0.87<br><.0001	0.81<br><.0001	0.66<br><.0001	0.64<br><.0001	0.67<br><.0001	0.56<br><.0001	0.58<br><.0001	0.51<br><.0001	0.03<br>0.39	0.40<br><.0001
PCB118			1	0.82<br><.0001	0.78<br><.0001	0.63<br><.0001	0.62<br><.0001	0.64<br><.0001	0.53<br><.0001	0.56<br><.0001	0.49<br><.0001	0.04<br>0.24	0.41<br><.0001
PCB138/158				1	0.98<br><.0001	0.89<br><.0001	0.86<br><.0001	0.81<br><.0001	0.71<br><.0001	0.73<br><.0001	0.64<br><.0001	0.003<br>0.93	0.40<br><.0001
PCB153					1	0.94<br><.0001	0.92<br><.0001	0.84<br><.0001	0.75<br><.0001	0.77<br><.0001	0.67<br><.0001	-0.01<br>0.73	0.40<br><.0001
PCB170						1	0.98<br><.0001	0.84<br><.0001	0.85<br><.0001	0.84<br><.0001	0.75<br><.0001	-0.01<br>0.70	0.34<br><.0001
PCB180							1	0.90	0.91	0.90	0.84	-0.01	0.35

								<.0001	<.0001	<.0001	<.0001	0.75	<.0001
PCB187								1	0.88<br><.0001	0.89<br><.0001	0.90<br><.0001	0.07<br>0.02	0.34<br><.0001
PCB194									1	0.96<br><.0001	0.94<br><.0001	0.006<br>0.85	0.29<br><.0001
PCB196/203										1	0.96<br><.0001	-0.004<br>0.89	0.31<br><.0001
PCB199											1	0.05<br>0.13	0.26<br><.0001
p,p’-DDE												1	0.05<br>0.12
Trans-Nonachlor													1

Pearson correlation coefficients of un-transformed concentrations.

Table S2: Demographic and basic characteristics by quartiles of selected analytes in controls[1]

	PCB153		***p,p'- DDE***	
	Q1<br>N=108	Q4<br>N=86	Q1<br>N= 114	Q4<br>N= 107
Maternal age (mean, std)	25.1 (5.2)[+]	32.4 (4.1)	28.0 (5.5)	29.0 (5.1)
Paternal age (mean, std)	27.9 (5.9)[+]	34.5 (4.4)	30.6 (6.4)	32.1 (5.9)
Child year of birth (mean, std)	2001 (0.9)	2001 (0.9)	2001 (1.0)	2001 (0.9)
# Prenatal visits (mean, std)	13.0 (3.8)	12.4 (3.2)	13.2 (4.7)	12.4 (3.7)
# Total live births (mean, std)	2.2 (1.2)	1.9 (0.9)	1.9 (0.9)	2.3 (1.1)
Child birth weight in grams (mean, std)	3423 (518)	3377 (539)	3459 (550)	3342 (535)
Child gestational age in days (mean, std)	277 (17.4)	275 (12.2)	283 (51)	275 (23)
Multiparous (n, %)	68 (63%)	53 (62%)	69 (61%)	73 (68%)
Maternal birth place (n, %)	*		*	
US	41 (38%)	32 (37%)	95 (83%)	6 (6%)
Mexico	62 (57%)	9 (10%)	8 (7%)	60 (56%)
Other	5 (5%)	45 (52%)	11 (10%)	41 (38%)

Maternal race/ethnicity (n, %)	*		*	
Non-Hispanic White	16 (15%)	31 (36%)	67 (59%)	3 (3%)
Asian	1 (1%)	31 (36%)	2 (2%)	29 (27%)
Black, PI & Other	3 (3%)	10 (12%)	10 (9%)	9 (8%)
Hispanic	87 (81%)	14 (16%)	34 (30%)	66 (62%)
Missing	1 (1%)	0	1 (1%)	0
Maternal education (n, %)	*		*	
Less than high school	54 (51%)	6 (7%)	14 (12%)	43 (40%)
High school	31 (29%)	17 (20%)	33 (29%)	29 (27%)
Some college/College degree	19 (18%)	42 (49%)	43 (38%)	29 (27%)
Post-graduate	3 (3%)	21 (24%)	23 (20%)	6 (6%)
Maternal age ≥35 years (n, %)	6 (6%)*	28 (33%)	11 (10%)	14 (13%)
Paternal age ≥35 years (n, %)	21 (19%)*	45 (52%)	28 (25%)*	40 (39%)
Insurance status at delivery (n, %)	*			
Self & other	8 (7%)	1 (1%)	4 (4%)	2 (2%)
Private insurance	23 (21%)	57 (66%)	66 (58%)	35 (33%)
Government program	77 (71%)	28 (33%)	44 (39%)	70 (65%)

Child sex[2] (n, %)	*			
Male	82 (76%)	76 (88%)	92 (81%)	86 (80%)
Female	26 (24%)	10 (12%)	22 (19%)	21 (20%)
Child preterm (<37 weeks) (n, %)	12 (12%)	6 (7%)	10 (9%)	12 (12%)
Child low birth weight (<2500g) (n, %)	2 (2%)	2 (2%)	6 (5%)	3 (3%)

[1]PCB153 and p,p-DDE chosen as representative of exposure to the class of PCBs and pesticides in this analysis, respectively, and due to high % detected above the LOD. Only quartiles 1 and 4 are shown for comparison/summary purposes. [2]Child sex was a matching factor to ASD cases, hence the unequal distribution.

[+]= significant difference according to t-test comparing Q1 vs Q4 on values of the variable.

*= significant difference according to Chi-square test comparing Q1 vs Q4 across categories of the covariate/demographic factor.

Table S3: Geometric means by diagnostic status

Compound	ASD<br>N=548	ID<br>N=181	GP<br>N=418
	Geometric mean (95% CI)		
PCBs			
PCB28	15.6 (14.0, 17.3)	17.4 (14.5, 21.0)	13.6 (12.0, 15.3)
PCB99	1.57 (1.49, 1.65)	1.25 (1.17, 1.34)	1.41 (1.33, 1.49)
PCB118	2.59 (2.45, 2.74)	1.94 (1.77, 2.12)	2.29 (2.14, 2.45)
PCB138/158	6.60 (6.18, 7.05)	4.69 (4.23, 5.20)	5.39 (4.99, 5.83)
PCB153	8.74 (8.19, 9.34)	3.91 (3.41, 4.48)	7.26 (6.74, 7.83)
PCB170	3.09 (2.89, 3.30)	2.10 (1.88, 2.35)	2.60 (2.41, 2.81)
PCB180	7.49 (6.99, 8.02)	4.79 (4.28, 5.35)	6.07 (5.60, 6.57)
PCB187	2.27 (2.10, 2.46)	1.51 (1.34, 1.70)	1.87 (1.70, 2.04)
PCB194	1.81 (1.70, 1.94)	1.27 (1.15, 1.40)	1.56 (1.44, 1.68)
PCB196/203	1.98 (1.85, 2.12)	1.35 (1.22, 1.49)	1.70 (1.57, 1.84)
PCB199	1.73 (1.60, 1.87)	1.13 (1.01, 1.26)	1.44 (1.32, 1.58)
Sum of above PCBs	62.4 (58.7, 66.4)	51.7 (46.5, 57.6)	54.6 (50.9, 58.5)
OC Pesticides			
p,p'-DDE	254.0 (232.4, 277.6)	304.6 (258.0, 359.7)	277.4 (247.3, 311.2)
Trans-nonachlor	5.11 (4.85, 5.39)	4.54 (4.12, 5.01)	4.76 (4.48, 5.05)

ASD= autism spectrum disorder cases; ID= intellectual disability (without ASD) group; GP= general population controls; PCBs= polychlorinated biphenyls; OC= organochlorine.

Table S4: Association between the top decile of organochlorine chemicals and risk of ASD and ID

	AOR (95% CI)[1]	
	**ASD vs. GP**	**ID vs. GP**
PCBs		
PCB28		
Lowest 10th percentile	1.0	1.0
10th-25th	1.07 (0.60, 1.88)	2.66 (1.12, 6.31)
IQR	1.23 (0.75, 2.03)	1.71 (0.76, 3.82)
75th-90th	1.06 (0.60, 1.88)	1.49 (0.61, 3.63)
Highest 10th percentile	1.70 (0.94, 3.07)	2.69 (1.10, 6.61)
PCB99		
Lowest 10th percentile	1.0	1.0
10th-25th	1.13 (0.66, 1.96)	1.77 (0.82, 3.83)
IQR	1.19 (0.75, 1.89)	1.71 (0.86, 3.41)
75th-90th	1.20 (0.68, 2.10)	2.14 (0.88, 5.23)
Highest 10th percentile	1.37 (0.74, 2.52)	1.24 (0.40, 3.85)
PCB118		
Lowest 10th percentile	1.0	1.0
10th-25th	0.85 (0.45, 1.59)	1.64 (0.73, 3.66)
IQR	1.20 (0.72, 2.01)	1.60 (0.80, 3.23)
75th-90th	1.18 (0.64, 2.16)	1.43 (0.58, 3.57)
Highest 10th percentile	0.78 (0.39, 1.56)	0.71 (0.20, 2.56)
PCB138/158		
Lowest 10th percentile	1.0	1.0

10th-25th	0.95 (0.51, 1.77)	1.39 (0.64, 3.04)
IQR	1.32 (0.76, 2.30)	2.07 (0.99, 4.33)
75th-90th	1.76 (0.91, 3.40)	3.70 (1.48, 9.25)
Highest 10th percentile	1.72 (0.85, 3.50)	2.11 (0.65, 6.85)
PCB153		
Lowest 10th percentile	1.0	1.0
10th-25th	1.00 (0.55, 1.81)	1.56 (0.77, 3.17)
IQR	1.28 (0.75, 2.16)	1.63 (0.83, 3.22)
75th-90th	2.02 (1.06, 3.85)	2.67 (1.11, 6.47)
Highest 10th percentile	1.50 (0.73, 3.06)	2.44 (0.81, 7.34)
PCB170		
Lowest 10th percentile	1.0	1.0
10th-25th	0.85 (0.47, 1.54)	0.98 (0.48, 1.97)
IQR	1.07 (0.65, 1.76)	0.90 (0.47, 1.70)
75th-90th	1.29 (0.68, 2.42)	1.37 (0.58, 3.25)
Highest 10th percentile	1.50 (0.74, 3.05)	1.57 (0.53, 4.61)
PCB180		
Lowest 10th percentile	1.0	1.0
10th-25th	1.33 (0.73, 2.45)	2.42 (1.14, 5.13)
IQR	1.30 (0.74, 2.28)	1.82 (0.86, 3.85)
75th-90th	1.70 (0.86, 3.35)	2.39 (0.89, 6.41)
Highest 10th percentile	1.87 (0.89, 3.94)	3.20 (1.02, 9.97)
PCB187		
Lowest 10th percentile	1.0	1.0

10th-25th	1.27 (0.71, 2.29)	2.34 (1.03, 5.29)
IQR	1.13 (0.67, 1.92)	2.03 (0.92, 4.48)
75th-90th	1.35 (0.71, 2.60)	2.89 (1.07, 7.86)
Highest 10th percentile	1.43 (0.68, 3.03)	3.54 (0.97, 12.9)
PCB194		
Lowest 10th percentile	1.0	1.0
10th-25th	1.17 (0.66, 2.07)	1.85 (0.89, 3.84)
IQR	1.11 (0.69, 1.78)	1.37 (0.71, 2.64)
75th-90th	1.29 (0.71, 2.76)	1.83 (0.75, 4.49)
Highest 10th percentile	1.41 (0.72, 2.76)	1.79 (0.55, 5.84)
PCB196/203		
Lowest 10th percentile	1.0	1.0
10th-25th	1.22 (0.68, 2.21)	2.51 (1.09, 5.79)
IQR	1.14 (0.66, 1.98)	2.58 (1.16, 5.74)
75th-90th	1.18 (0.61, 2.29)	3.22 (1.19, 8.71)
Highest 10th percentile	1.62 (0.78, 3.34)	3.21 (0.90, 11.4)
PCB199		
Lowest 10th percentile	1.0	1.0
10th-25th	1.50 (0.74, 3.04)	3.11 (1.10, 8.82)
IQR	1.44 (0.73, 2.83)	2.95 (1.06, 8.23)
75th-90th	1.34 (0.61, 2.93)	4.03 (1.23, 13.3)
Highest 10th percentile	1.81 (0.77, 4.24)	2.67 (0.56, 12.7)
Sum of above PCBs		
Lowest 10th percentile	1.0	1.0

10th-25th	0.47 (0.26, 0.86)	1.00 (0.46, 2.18)
IQR	0.71 (0.44, 1.17)	1.10 (0.57, 2.14)
75th-90th	0.97 (0.54, 1.73)	1.41 (0.63, 3.18)
Highest 10th percentile	0.87 (0.46, 1.66)	1.44 (0.57, 3.66)
OC pesticides		
Trans-Nonachlor		
Lowest 10th percentile	1.0	1.0
10th-25th	3.06 (1.66, 5.64)	1.85 (0.86, 4.00)
IQR	1.82 (1.07, 3.11)	1.12 (0.57, 2.21)
75th-90th	1.74 (0.94, 3.23)	1.40 (0.62, 3.17)
Highest 10th percentile	1.77 (0.91, 3.46)	1.26 (0.49, 3.24)
p,p-DDE		
Lowest 10th percentile	1.0	1.0
10th-25th	1.12 (0.64, 1.96)	1.37 (0.56, 3.37)
IQR	1.37 (0.84, 2.21)	1.96 (0.90, 4.24)
75th-90th	1.29 (0.71, 2.34)	1.35 (0.54, 3.25)
Highest 10th percentile	0.51 (0.25, 1.04)	1.29 (0.49, 3.43)

[1]Adjusted as in Tables 3 and 4 for matching factors (child sex, month and year of birth), maternal age, weight at sample collection, education, race/ethnicity, parity.

Table S5: Stratified and Subgroup Analyses of Prenatal PCB and OC Pesticide levels in association with ASD (vs GP controls)

	Stratified analyses				*ASD phenotypic subgroup analyses*	
	Males n=791 (446 cases)	Females n=172 (99 cases)	Non-Hispanic White n=330 (192 cases)	Hispanic n=415 (218 cases)	ASD with ID n= 705 (287 cases)	ASD without ID n=676 (258 cases)
	**Adjusted OR (95% CI)[1]**					
PCBs						
**PCB28**						
Q1	1.0	1.0	1.0	1.0	1.0	1.0
Q2	1.31 (0.85, 2.02)	0.74 (0.25, 2.22)	1.38 (0.67, 2.82)	1.29 (0.72, 2.31)	1.32 (0.83, 2.09)	0.96 (0.58, 1.59)
Q3	1.29 (0.84, 1.97)	1.17 (0.38, 3.58)	1.43 (0.73, 2.78)	1.10 (0.61, 1.99)	1.13 (0.71, 1.80)	1.32 (0.82, 2.13)
Q4	1.43 (0.94, 2.17)	0.86 (0.29, 2.53)	1.95 (0.98, 3.87)	1.13 (0.65, 1.98)	1.23 (0.79, 1.94)	1.31 (0.81, 2.11)
**PCB99**						
Q1	1.0	1.0	1.0	1.0	1.0	1.0

Q2	1.04 (0.65, 1.65)	1.82 (0.66, 5.01)	0.84 (0.36, 1.95)	1.12 (0.66, 1.92)	1.11 (0.68, 1.79)	1.19 (0.69, 2.05)
Q3	1.05 (0.67, 1.66)	1.44 (0.52, 3.98)	1.60 (0.74, 3.48)	0.77 (0.45, 1.32)	0.99 (0.62, 1.60)	1.24 (0.74, 2.09)
Q4	1.04 (0.64, 1.69)	2.68 (0.79, 9.11)	1.59 (0.75, 3.38)	1.50 (0.68, 3.28)	1.03 (0.61, 1.74)	1.42 (0.82, 2.45)
**PCB118**						
Q1	1.0	1.0	1.0	1.0	1.0	1.0
Q2	1.29 (0.82, 2.04)	1.29 (0.46, 3.58)	1.29 (0.60, 2.78)	1.52 (0.89, 2.60)	1.47 (0.89, 2.41)	1.06 (0.63, 1.79)
Q3	1.34 (0.83, 2.16)	1.92 (0.67, 5.54)	1.36 (0.62, 2.96)	1.44 (0.80, 2.57)	1.59 (0.95, 2.65)	1.15 (0.67, 1.98)
Q4	1.07 (0.64, 1.79)	1.75 (0.52, 5.82)	1.47 (0.67, 3.26)	1.18 (0.54, 2.54)	1.22 (0.70, 2.14)	1.06 (0.59, 1.87)
**PCB138/158**						
Q1	1.0	1.0	1.0	1.0	1.0	1.0
Q2	1.22 (0.77, 1.92)	2.49 (0.84, 7.39)	0.86 (0.38, 1.99)	1.85 (1.09, 3.15)	1.44 (0.88, 2.36)	1.26 (0.73, 2.14)
Q3	1.22 (0.75, 1.98)	1.80 (0.60, 5.41)	1.25 (0.53, 2.92)	1.32 (0.74, 2.38)	1.67 (1.00, 2.80)	1.02 (0.58, 1.78)
Q4	*1.70 (1.00, 2.89)*	1.92 (0.48, 7.75)	1.61 (0.66, 3.92)	1.85 (0.84, 4.07)	1.71 (0.95, 3.07)	1.89 (1.03, 3.46)
**PCB153**						
Q1	1.0	1.0	1.0	1.0	1.0	1.0
Q2	1.27 (0.80, 1.99)	1.12 (0.38, 3.32)	0.90 (0.35, 2.31)	1.54 (0.93, 2.54)	1.26 (0.77, 2.03)	1.41 (0.82, 2.44)

Q3	1.03 (0.63, 1.70)	2.13 (0.73, 6.20)	1.12 (0.43, 2.92)	1.47 (0.81, 2.68)	1.27 (0.76, 2.14)	1.24 (0.70, 2.20)
Q4	1.73 (0.99, 3.02)	1.48 (0.38, 5.70)	1.43 (0.53, 3.90)	1.72 (0.74, 3.99)	1.64 (0.91, 2.96)	2.20 (1.17, 4.16)
**PCB170**						
Q1	1.0	1.0	1.0	1.0	1.0	1.0
Q2	1.02 (0.63, 1.63)	1.94 (0.67, 5.63)	0.38 (0.15, 1.01)	1.75 (1.02, 2.98)	1.28 (0.77, 2.11)	0.98 (0.57, 1.70)
Q3	0.98 (0.60, 1.62)	2.36 (0.71, 7.82)	0.49 (0.19, 1.30)	1.63 (0.88, 3.02)	1.47 (0.87, 2.51)	0.87 (0.49, 1.54)
Q4	1.23 (0.70, 2.17)	3.94 (0.88, 17.7)	0.68 (0.24, 1.97)	1.31 (0.57, 3.04)	1.61 (0.87, 2.99)	1.36 (0.71, 2.59)
**PCB180**						
Q1	1.0	1.0	1.0	1.0	1.0	1.0
Q2	0.95 (0.60, 1.50)	1.20 (0.43, 3.33)	0.40 (0.16, 1.03)	1.28 (0.77, 2.12)	0.93 (0.57, 1.50)	1.09 (0.64, 1.87)
Q3	1.09 (0.67, 1.78)	1.29 (0.43, 3.82)	0.69 (0.26, 1.83)	1.44 (0.79, 2.60)	1.21 (0.72, 2.02)	1.14 (0.65, 2.01)
Q4	1.35 (0.77, 2.37)	2.08 (0.53, 8.10)	0.71 (0.24, 2.07)	1.51 (0.67, 3.44)	1.51 (0.83, 2.75)	1.55 (0.82, 2.95)
**PCB187**						
Q1	1.0	1.0	1.0	1.0	1.0	1.0
Q2	0.76 (0.48, 1.22)	1.67 (0.57, 4.87)	0.60 (0.25, 1.45)	1.15 (0.68, 1.95)	0.86 (0.52, 1.43)	0.92 (0.53, 1.58)
Q3	1.15 (0.71, 1.86)	1.48 (0.51, 4.29)	1.18 (0.48, 2.92)	1.29 (0.74, 2.27)	1.32 (0.80, 2.18)	1.12 (0.65, 1.93)

Q4	1.22 (0.69, 2.16)	1.58 (0.43, 5.80)	0.93 (0.35, 2.48)	1.76 (0.71, 4.35)	1.25 (0.69, 2.28)	1.39 (0.74, 2.61)
**PCB194**						
Q1	1.0	1.0	1.0	1.0	1.0	1.0
Q2	1.01 (0.64, 1.61)	0.96 (0.33, 2.79)	1.07 (0.40, 2.83)	1.22 (0.72, 2.07)	0.80 (0.49, 1.31)	1.27 (0.75, 2.14)
Q3	1.02 (0.63, 1.66)	1.38 (0.48, 3.98)	1.43 (0.54, 3.81)	0.85 (0.47, 1.54)	1.20 (0.73, 1.97)	0.97 (0.56, 1.69)
Q4	1.20 (0.69, 2.09)	1.86 (0.55, 6.26)	1.52 (0.50, 4.58)	1.28 (0.57, 2.90)	1.18 (0.66, 2.10)	1.36 (0.74, 2.52)
**PCB196/203**						
Q1	1.0	1.0	1.0	1.0	1.0	1.0
Q2	0.78 (0.48, 1.25)	1.04 (0.36, 2.98)	0.64 (0.25, 1.66)	0.95 (0.56, 1.62)	0.56 (0.33, 0.95)	1.15 (0.67, 1.98)
Q3	1.03 (0.64, 1.65)	1.24 (0.42, 3.70)	0.94 (0.39, 2.27)	1.10 (0.61, 1.98)	1.15 (0.70, 1.90)	0.95 (0.55, 1.66)
Q4	1.05 (0.60, 1.84)	1.72 (0.47, 6.34)	1.06 (0.38, 2.98)	0.98 (0.43, 2.25)	1.04 (0.57, 1.88)	1.26 (0.67, 2.36)
**PCB199**						
Q1	1.0	1.0	1.0	1.0	1.0	1.0
Q2	0.80 (0.51, 1.28)	1.96 (0.67, 5.73)	0.83 (0.33, 2.10)	0.91 (0.54, 1.52)	0.81 (0.50, 1.33)	1.10 (0.64, 1.89)
Q3	1.09 (0.67, 1.76)	1.58 (0.51, 4.87)	1.19 (0.48, 2.95)	1.16 (0.64, 2.09)	1.23 (0.75, 2.04)	1.11 (0.64, 1.93)
Q4	1.02 (0.58, 1.81)	1.91 (0.51, 7.12)	1.03 (0.36, 2.93)	0.86 (0.36, 2.02)	1.00 (0.54, 1.85)	1.22 (0.65, 2.29)

**Sum of above PCBs**						
Q1	1.0	1.0	1.0	1.0	1.0	1.0
Q2	0.95 (0.60, 1.50)	2.80 (0.92, 8.56)	1.66 (0.74, 3.71)	1.08 (0.62, 1.88)	1.09 (0.67, 1.76)	1.07 (0.63, 1.83)
Q3	0.90 (0.56, 1.45)	1.91 (0.65, 5.61)	1.37 (0.61, 3.12)	0.72 (0.40, 1.31)	0.74 (0.44, 1.25)	1.33 (0.78, 2.26)
Q4	1.33 (0.82, 2.16)	1.72 (0.55, 5.36)	2.06 (0.90, 4.74)	1.32 (0.69, 2.51)	1.28 (0.77, 2.12)	1.47 (0.84, 2.57)
OC Pesticides						
**p,p-DDE**						
Q1	1.0	1.0	1.0	1.0	1.0	1.0
Q2	1.18 (0.78, 1.77)	2.86 (1.05, 7.82)	1.16 (0.70, 1.92)	1.44 (0.73, 2.84)	1.31 (0.84, 2.03)	1.48 (0.94, 2.34)
Q3	1.11 (0.71, 1.74)	1.51 (0.53, 4.32)	1.37 (0.69, 2.69)	1.17 (0.61, 2.25)	1.06 (0.65, 1.71)	1.38 (0.83, 2.31)
Q4	0.87 (0.53, 1.44)	1.29 (0.42, 4.03)	0.95 (0.20, 4.49)	0.96 (0.49, 1.89)	0.84 (0.50, 1.42)	1.11 (0.62, 1.99)
**Trans-Nonachlor**	1.0	1.0	1.0	1.0	1.0	1.0
Q1	0.89 (0.57, 1.38)	1.16 (0.44, 3.10)	1.25 (0.60, 2.59)	0.94 (0.54, 1.64)	0.76 (0.48, 1.21)	1.14 (0.69, 1.87)
Q2	0.78 (0.50, 1.22)	1.37 (0.46, 4.14)	0.63 (0.31, 1.30)	1.00 (0.54, 1.86)	0.76 (0.47, 1.22)	0.93 (0.55, 1.55)

Q3	0.78 (0.49, 1.26)	1.05 (0.37, 2.95)	1.13 (0.54, 2.33)	1.02 (0.52, 2.00)	0.76 (0.46, 1.25)	0.96 (0.57, 1.62)
Q4						

Estimates stratified by maternal age not shown given small n in the advanced maternal age stratum. [1]Adjusted as in primary tables 3 and 4, for matching factors (child sex, month and year of birth), maternal age, race/ethnicity, education, weight at sample collection, and parity.

Table S6: Sensitivity Analyses of Prenatal PCB and OC Pesticide levels in association with ASD (vs GP controls)

	CLR matched pairs[1] n=788	Uncensored laboratory data[2] n=963	Multiple imputation[3] n=963	Exclusion of ID to ASD group[4] n=831
	Adjusted OR (95% CI)[5]			
**PCB28**				
Q1	1.0	1.0	1.0	1.0
Q2	1.41 (0.85, 2.33)	1.15 (0.77, 1.71)	1.94 (1.31, 2.88)	1.05 (0.68, 1.60)
Q3	1.67 (0.97, 2.87)	1.20 (0.81, 1.77)	1.43 (0.96, 2.13)	1.15 (0.75, 1.74)
Q4	1.56 (0.86, 2.83)	1.26 (0.86, 1.85)	1.94 (1.31, 2.87)	1.20 (0.80, 1.82)
**PCB99**				
Q1	1.0	1.0	1.0	1.0
Q2	1.17 (0.70, 1.93)	0.95 (0.63, 1.45)	0.79 (0.33, 1.91)	1.09 (0.69, 1.72)
Q3	1.30 (0.77, 2.19)	1.07 (0.71, 1.59)	1.20 (0.56, 2.58)	1.17 (0.76, 1.81)
Q4	1.17 (0.66, 2.07)	1.01 (0.64, 1.59)	0.95 (0.44, 2.05)	1.17 (0.72, 1.88)

**PCB118**				
Q1	1.0	1.0	1.0	1.0
Q2	1.19 (0.73, 1.95)	1.32 (0.87, 2.00)	1.01 (0.66, 1.57)	1.28 (0.82, 1.99)
Q3	1.41 (0.84, 2.36)	1.40 (0.91, 2.16)	1.17 (0.75, 1.84)	1.33 (0.84, 2.11)
Q4	1.07 (0.61, 1.86)	1.17 (0.73, 1.87)	1.00 (0.64, 1.57)	1.14 (0.70, 1.88)
**PCB138_158**				
Q1	1.0	1.0	1.0	1.0
Q2	1.19 (0.73, 1.94)	1.39 (0.92, 2.10)	1.34 (0.88, 2.05)	1.15 (0.73, 1.81)
Q3	1.21 (0.73, 2.00)	1.34 (0.87, 2.07)	1.25 (0.80, 1.95)	1.16 (0.73, 1.85)
Q4	1.60 (0.90, 2.83)	1.79 (1.10, 2.92)	1.57 (0.96, 2.59)	1.61 (0.95, 2.72)
**PCB153**				
Q1	1.0	1.0	1.0	1.0
Q2	1.11 (0.66, 1.85)	1.38 (0.92, 2.09)	1.11 (0.73, 1.70)	1.12 (0.71, 1.75)
Q3	1.18 (0.70, 1.97)	1.28 (0.82, 2.00)	1.05 (0.67, 1.67)	1.14 (0.71, 1.84)
Q4	1.64 (0.91, 2.96)	1.89 (1.14, 3.13)	1.49 (0.88, 2.50)	1.88 (1.09, 3.24)
**PCB170**				

Q1	1.0	1.0	1.0	1.0
Q2	1.14 (0.69, 1.89)	1.16 (0.76, 1.76)	1.02 (0.65, 1.62)	1.04 (0.66, 1.64)
Q3	1.06 (0.62, 1.82)	1.17 (0.75, 1.84)	1.04 (0.65, 1.67)	1.05 (0.65, 1.70)
Q4	1.31 (0.71, 2.42)	1.49 (0.88, 2.51)	1.34 (0.78, 2.29)	1.44 (0.82, 2.51)
**PCB180**				
Q1	1.0	1.0	1.0	1.0
Q2	0.93 (0.57, 1.53)	1.04 (0.69, 1.56)	0.85 (0.55, 1.29)	0.88 (0.56, 1.36)
Q3	1.06 (0.64, 1.75)	1.20 (0.77, 1.85)	1.03 (0.66, 1.62)	1.01 (0.63, 1.62)
Q4	1.43 (0.80, 2.58)	1.53 (0.92, 2.55)	1.08 (0.63, 1.83)	1.53 (0.88, 2.66)
**PCB187**				
Q1	1.0	1.0	1.0	1.0
Q2	0.77 (0.46, 1.29)	1.19 (0.78, 1.81)	0.82 (0.49, 1.38)	0.80 (0.51, 1.26)
Q3	1.21 (0.72, 2.04)	1.44 (0.93, 2.24)	1.00 (0.58, 1.72)	1.14 (0.72, 1.80)
Q4	1.33 (0.73, 2.42)	1.58 (0.94, 2.66)	1.04 (0.60, 1.81)	1.39 (0.80, 2.39)
**PCB194**				
Q1	1.0	1.0	1.0	1.0

Q2	0.92 (0.54, 1.55)	1.03 (0.68, 1.57)	0.98 (0.55, 1.73)	0.93 (0.59, 1.46)
Q3	0.93 (0.55, 1.56)	1.17 (0.74, 1.83)	1.34 (0.78, 2.31)	1.00 (0.63, 1.59)
Q4	1.21 (0.67, 2.18)	1.33 (0.79, 2.23)	1.32 (0.74, 2.37)	1.32 (0.78, 2.23)
**PCB196_203**				
Q1	1.0	1.0	1.0	1.0
Q2	0.68 (0.40, 1.14)	0.97 (0.64, 1.49)	0.89 (0.55, 1.43)	0.73 (0.46, 1.16)
Q3	0.93 (0.56, 1.54)	1.12 (0.72, 1.73)	1.16 (0.71, 1.91)	0.97 (0.62, 1.54)
Q4	1.22 (0.67, 2.22)	1.24 (0.74, 2.07)	1.26 (0.75, 2.11)	1.20 (0.70, 2.05)
**PCB199**				
Q1	1.0	1.0	1.0	1.0
Q2	0.78 (0.47, 1.30)	0.95 (0.62, 1.45)	0.93 (0.60, 1.43)	0.79 (0.50, 1.24)
Q3	0.98 (0.58, 1.66)	1.26 (0.81, 1.97)	1.36 (0.85, 2.16)	1.03 (0.65, 1.63)
Q4	1.14 (0.63, 2.06)	1.19 (0.70, 2.02)	1.11 (0.67, 1.86)	1.15 (0.66, 1.98)
**Sum of above PCBs**			*	
Q1	1.0	1.0	1.0	1.0
Q2	1.08 (0.65, 1.80)	1.08 (0.71, 1.63)	1.37 (0.77, 2.44)	0.94 (0.60, 1.47)

Q3	1.01 (0.59, 1.73)	1.01 (0.66, 1.55)	1.48 (0.82, 2.70)	1.05 (0.67, 1.64)
Q4	1.55 (0.83, 2.89)	1.36 (0.87, 2.12)	1.71 (0.91, 3.23)	1.34 (0.84, 2.14)
**p,p-DDE**				
Q1	1.0	1.0	1.0	1.0
Q2	1.40 (0.92, 2.11)	1.35 (0.93, 1.96)	1.34 (0.90, 2.00)	1.41 (0.95, 2.10)
Q3	1.11 (0.70, 1.77)	1.16 (0.77, 1.74)	1.11 (0.72, 1.71)	1.23 (0.79, 1.90)
Q4	0.79 (0.46, 1.37)	0.90 (0.57, 1.42)	0.80 (0.50, 1.30)	0.95 (0.58, 1.54)
**Trans-Nonachlor**				
Q1	1.0	1.0	1.0	1.0
Q2	0.86 (0.53, 1.40)	1.20 (0.80, 1.79)	0.88 (0.48, 1.63)	0.93 (0.60, 1.42)
Q3	1.00 (0.62, 1.64)	1.06 (0.70, 1.62)	0.73 (0.39, 1.37)	0.96 (0.62, 1.48)
Q4	0.96 (0.57, 1.61)	1.02 (0.66, 1.57)	0.93 (0.50, 1.74)	0.94 (0.60, 1.48)

N listed for each column includes ASD cases + GP controls.

[1]CLR= conditional logistic regression; 394 pairs of ASD cases and GP controls, matched on sex, year and month of birth, were used in these analyses. [2]Original concentrations received from laboratory analysis of these chemicals included censoring of values with small volumes, due to potential bias. These analyses include all concentration data, even those with small sample volumes, in order to examine potential differences in censored vs uncensored data (and as a comparison to the primary method of replacing values <LOD with

LOD/√2). [3]Results using SAS Proc MI, as described in the text, for those individuals with values <LOD (as a comparison to the primary method of replacing values <LOD with LOD/√2). [4]These results exclude the n=132 cases who were originally identified as having ID/developmental delay through the California DDS system, but were classified as ASD in our study following expert review of DDS records.[5]All adjusted models shown in this table include: maternal race/ethnicity (Non-Hispanic White, Asian, Black/Pacific Islander/or other, Hispanic, or missing), maternal weight at time of sample collection (quartiles), parity (multi- vs primiparous), and maternal education (<high school, high school, college, graduate). CLR matched pairs model (results column 1) is stratified by matched pairs, while the other models include adjustment for the matching factors in logistic regression models. Models additionally adjusted for the PCs overall produced similar estimates.

Tests of trend were non-significant. *Validity of MI model fit questionable; CIs may not be valid.
